# Ovarian cancer in younger *vs* older women: a population-based analysis

**DOI:** 10.1038/sj.bjc.6603457

**Published:** 2006-10-31

**Authors:** J K Chan, R Urban, M K Cheung, K Osann, A Husain, N N Teng, D S Kapp, J S Berek, G S Leiserowitz

**Affiliations:** 1 Division of Gynecologic Oncology, 875 Blake Wilbur Drive, Stanford, CA 94305, USA; 2 Division of Radiation Oncology, 875 Blake Wilbur Drive, Stanford, CA 94305, USA; 3 Stanford Cancer Center, 875 Blake Wilbur Drive, Stanford, CA 94305, USA; 4 Division of Hematology/Oncology, Chao Family Comprehensive Cancer Center, University of California, Irvine – Medical Center, 101 The City Drive, Orange, CA 92868, USA; 5 Division of Gynecology Oncology, University of California, Davis Cancer Center, 4501 X Street, Sacramento, CA, 95817, USA

**Keywords:** ovarian cancer, young, uterine-sparing, survival

## Abstract

To compare the clinico-pathologic prognostic factors and survival of younger *vs* older women diagnosed with epithelial ovarian cancer. Demographic, clinico-pathologic, treatment, and surgery information were obtained from patients with ovarian cancer from the Surveillance, Epidemiology, and End Results Program from 1988 to 2001 and analysed using Kaplan–Meier estimates. Of 28 165 patients, 400 were <30 years (very young), 11 601 were 30–60 (young), and 16 164 were >60 (older) years of age. Of the very young, young, and older patients, 261 (65.3%), 4664 (40.2%), and 3643 (22.5%) had stage I–II disease, respectively (*P*<0.001). Across all stages, very young women had a significant survival advantage over the young and older groups with 5-year disease-specific survival estimates at 78.8% *vs* 58.8 and 35.3%, respectively (*P*<0.001). This survival difference between the age groups persists even after adjusting for race, stage, grade, and surgical treatment. Reproductive age (16–40 years) women with stage I–II epithelial ovarian cancer who received uterine-sparing procedures had similar survivals compared to those who underwent standard surgery (93.3% *vs* 91.5%, *P*=0.26). Younger women with epithelial ovarian cancer have a survival advantage compared to older patients.

As the most lethal of the gynaecologic malignancies, ovarian cancer remains the fifth most common cause of cancer-related death for women in the United States. An estimated 25 580 new cases of ovarian cancer were diagnosed in the United States in the year 2004, with 16 090 deaths associated with this disease (Jemal *et al*, 2004). Despite advances in surgical and systemic treatment, the 5-year survival of advanced stage patients with ovarian cancer remains below 30% (Nguyen *et al*, 1993; Parker *et al*, 1996; Jemal *et al*, 2004; Ries *et al*, 2004). However, invasive epithelial ovarian cancer is largely a disease of postmenopausal women with reproductive-age women comprising only 20% of all patients (Smedley and Sikora, 1985; Swenerton *et al*, 1985; Plaxe *et al*, 1993; Rodriguez *et al*, 1994). Previous studies on young patients with ovarian cancer have reported survival rates as high as 75% across all stages compared to 40% in the overall population.

Some reports have indicated that younger age is not an independent prognostic factor for improved survival, suggesting that the survival advantage of the younger patients may be attributed to the increased frequency of early-stage, lower grade disease, and tumours of low malignant potential (Massi *et al*, 1996; Duska *et al*, 1999). Nonetheless, several other published studies have specified age as a significant prognostic factor, with older patients faring less well than younger women (Smedley and Sikora, 1985; Swenerton *et al*, 1985; Yancik *et al*, 1986; Omura *et al*, 1991; Gloecker-Ries, 1993; Ries, 1993; Thigpen *et al*, 1993; Kosary, 1994; Rodriguez *et al*, 1994; Lee *et al*, 1999; Brun *et al*, 2000; Schildkraut *et al*, 2000; Barnholtz-Sloan *et al*, 2003; Chan *et al*, 2003; O’Malley *et al*, 2003).

Many of these studies are institutional-based analyses comprising a small number of patients. Furthermore, most reports originated from academic institutions and clinical trials data with associated biases and outcomes that may not reflect the general population (Averette *et al*, 1995; Barnholtz-Sloan *et al*, 2003; Chan *et al*, 2003). Prior studies that have used data from national registries included information collected from 1973 to 1997 (Yancik *et al*, 1986; Ries, 1993; Kosary, 1994; Barnholtz-Sloan *et al*, 2003). Since that time, there have been important changes that have affected the care of ovarian cancer patients. In addition, many studies have not thoroughly evaluated important clinico-pathologic prognostic factors. Consequently, there are limited population-based studies with detailed surgical staging and pathology data that have evaluated the demographics, clinico-pathologic, and survival outcomes of younger women diagnosed with epithelial ovarian cancer.

In this study of over 28 000 epithelial ovarian cancer patients, we propose to determine if young age is an independent factor associated with improved survival and identify the clinico-pathologic prognostic factors associated with the survival of younger patients.

## MATERIALS AND METHODS

Demographic, clinico-pathological, treatment, and survival information on 28 165 women diagnosed with primary epithelial ovarian cancer during the period from 1988 to 2001 were extracted from the Surveillance, Epidemiology and End Results (SEER) database of the United States National Cancer Institute. All patients with low malignant potential tumours of the ovary were excluded from our analysis. Patients diagnosed with germ cell, sarcomas, and sex cord stromal histologic cell types were excluded. Data are reported from 12 population-based registries that represent approximately 14% of the US population including San Francisco-Oakland, Connecticut, metropolitan Detroit, Hawaii, Iowa, New Mexico, Seattle (Puget Sound), Utah, metropolitan Atlanta, Alaska, San Jose-Monterey, and Los Angeles (Hankey *et al*, 1999).

To further define the young patients with epithelial ovarian cancer, we divided our young patient population into two subgroups, <30 and 30–60, which we arbitrarily called the very young and young groups *vs* the older group >60 years. The race classifications of the SEER program were categorised into four groups: White, Black, Asian, and Others. Asians were defined as Chinese, Japanese, Korean, Vietnamese, and Filipina. All other race and ethnicity classifications were defined as Others. Surgical treatment was classified as either absent, uterine-sparing, or standard. Uterine-sparing surgeries were defined as surgeries that did not include a hysterectomy whereas standard surgeries included those reported to have had a hysterectomy and/or radical debulking.

**Figure 1:**
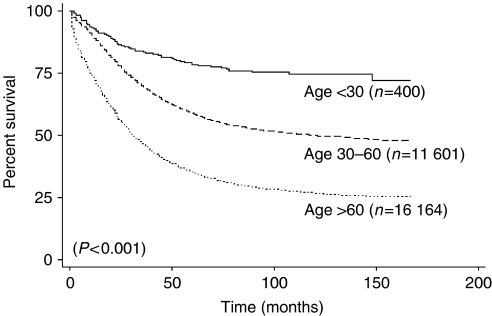
Kaplan–Meier disease-specific survival of patients based on age at diagnosis.

To analyse distribution patterns in the study cohort and to determine 5-year disease-specific survival, *χ*^2^ tests, and Kaplan–Meier analysis with log-rank tests were performed for the age groups defined above. The distributions of demographic, clinical, histologic, and treatment characteristics were compared using the *χ*^2^ test. Survival analysis was performed using the Kaplan–Meier estimates of survival probability across all three age groups, and the Cox-proportional hazards model was used to identify independent predictors of survival (Figure 1). The outcome of interest was death from ovarian cancer (disease-specific survival) and time to death was censored in women who died from causes other than ovarian cancer. Statistical analysis was performed using the Intercooled STATA 8.0 program (College Station, TX, USA). A two-sided *P*-value <0.05 was considered statistically significant.

## RESULTS

28 165 patients were diagnosed with primary epithelial ovarian cancer from 1988 to 2001. We divided our young patient population into two groups: the very young group (<30 years) consisting of 400 women and the young group (30–60 years) with 11 601 patients. To provide a means for comparison, 16 164 older patients (>60 years) were used as controls. The median age of the young and very young patients was 26 (range: 12–29) and 50 years (range: 30–60), respectively. In contrast, the median age for the control group was 72 years (range: 61–101).

Although the majority of patients in the three age groups were White, they comprised a significantly higher proportion of older patients as compared to younger patients (78.3% for very young, 83.6% for young, and 89.2% for the older group; *P*<0.001). In contrast, Black subjects comprised 7.8% of the very young, 6.1% young, and only 5.6% of the older age group (*P*<0.001). Similarly, other minority groups such as Asians and Hispanics are more heavily represented in the younger age groups (Table 1).

The very young and young patients were more likely to undergo a primary surgical procedure at 96.5 and 93.1% compared to 73.0% in the older age group (Table 2). More specifically, the very young patients are more likely to receive a uterine-preserving procedure compared to the older age groups (71.2 *vs* 14.1 *vs* 15.6%, respectively; *P*<0.001). Of those who underwent standard surgery, 4628 (24.8%), 1715 (9.2%), 7334 (39.2%), and 5018 (26.8%) had stage I, II, III, and IV disease. Moreover, of the women who had uterine-sparing procedures, 1393 (32.5%) had stage I disease, 306 (7.1%) stage II, 1343 (31.3%) stage III, and 1243 (29.0%) had stage IV disease.

In the overall study group, 22.3% had stage I, 8.2% stage II, 35.9% stage III, and 33.7% had stage IV disease. Younger patients were diagnosed with significantly more early-stage disease than older patients. In fact, 65.3 and 40.2% of the very young and young age groups presented with stage I–II disease compared to only 22.5% in the >60 age group (*P*<0.001). Younger patients were also more likely to be diagnosed with grade 1 disease with 33.8 and 12.0% of those in the very young and young group having well-differentiated tumours compared to only 5.4% in the older patients (*P*<0.001). In the entire study group, 1411 (4.7%) were clear cell cancers of the ovary. There were no significant differences in the various histologic cell types amongst the three age groups.

The overall 5-year disease-specific survivals of women <30, 30–60, and >60 age groups were 78.8, 58.8, and 35.3%, respectively (*P*<0.001; Table 3). Of those with early-stage disease, the very young and young patients had a significant survival advantage at 89.7 and 88.8% compared to 74.5% in the older age group (*P*<0.001). Similarly, older women with advanced-stage disease had poorer survival at only 22.1% compared to 55.7 and 36.9% in the very young and younger women (*P*<0.001). Younger age continues to portend for a better prognosis across ethnic, histologic cell types, and year of diagnosis. Furthermore, younger age (*P*<0.001), more recent year of diagnosis (*P*<0.001), non-clear cell epithelial histologic cell types (*P*<0.001), earlier stage of disease (Stage I *vs* Stage II *vs* Stage III *vs* Stage IV, *P*<0.001), lower grade (*P*<0.001), and surgical treatment (no surgery *vs* any surgery, *P*<0.001) remained as independent prognostic factors for improved survival (Table 4). When data for patients <30 years, 30–60 and >60 years were analysed, similar results confirming the importance of age as an independent prognostic factor groups were found (hazard ratio=1.20, 95% CI=1.13–1.28; *P*<0.001).

**Figure 2:**
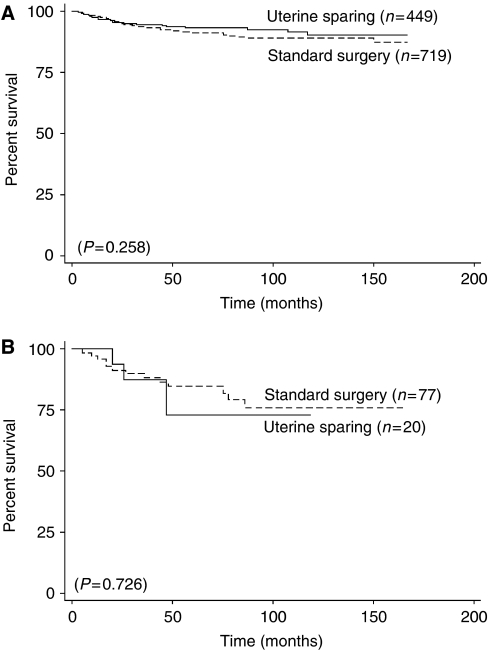
Kaplan–Meier disease-specific survival of reproductive age patients (age 16–40 years) with stage I–II (**A**) epithelial and (**B**) clear cell ovarian cancer.

Interestingly, reproductive age women, age 16–40, with stage I–II epithelial ovarian cancer who underwent uterine-sparing surgical procedures (*n*=435) had similar rates of survival compared to their counterparts (*n*=620) who underwent standard surgery (93.3 *vs* 91.5%; *P*=0.26). Furthermore, there were no significant survival differences associated with these two surgical approaches in those with stage I–II non-clear cell epithelial tumours (94.0% (*n*=449) *vs* 92.4% (*n*=719); *P*=0.41, Figure 2A) or stage I–II clear cell cancers (72.9% (*n*=20) *vs* 84.8% (*n*=77); *P*=0.73, Figure 2B). Even in patients with stage IIIC disease, we were unable to demonstrate a statistically significant survival difference (49.6% (*n*=27) *vs* 68.9% (*n*=258), respectively; *P*=0.22).

## DISCUSSION

Ovarian cancer is primarily a disease of postmenopausal women. Previous studies have shown that only 3–17% of patients with ovarian cancer were age <40 years (Plaxe *et al*, 1993; Rodriguez *et al*, 1994). The reported prognostic significance of age in female cancers has been inconsistent. Younger age has been shown to be a poor prognosticator in breast cancer. Recently, Maggard and others have demonstrated that younger (<35) women with breast cancer have a poorer prognosis owing to higher stage and grade of disease at presentation (Yildirim *et al*, 2000; Biffl *et al*, 2001; Xiong *et al*, 2001; Dubsky *et al*, 2002; Kothari *et al*, 2002; Love *et al*, 2002; Maggard *et al*, 2003). However, studies on the prognostic implications of age and ovarian cancer are inconclusive. Although most reports have shown that younger women with ovarian cancer have an improved outcome compared to older women and have lower stage and more well-differentiated tumours (Smedley and Sikora, 1985; Plaxe *et al*, 1993; Thigpen *et al*, 1993; Rodriguez *et al*, 1994; Chan *et al*, 2003), others have found that age is not an independent prognostic factor after adjusting for tumour stage and grade (Massi *et al*, 1996; Duska *et al*, 1999). Moreover, the majority of these previous studies on ovarian cancer are based on single institution experiences, which contain inherent biases such as patient selection (Plaxe *et al*, 1993; Duska *et al*, 1999; Chan *et al*, 2003). In addition, owing to the low prevalence of young patients diagnosed with invasive ovarian cancer, these studies have also been limited by a small number of patients, inclusion of low malignant potential tumours, germ cell or sex cord stromal tumours, and unstaged cancers. European analyses on ovarian cancer incidence rates include studies carried out by Smedley and Sikora (1985), La Vecchia *et al* (1992), dos Santos Silva and Swerdlow (1995), Koper *et al* (1996), Nelson *et al* (1999). Given the limitations from prior reports, we proposed to perform a large population-based study to evaluate the clinico-pathologic characteristics between younger and older patients with epithelial ovarian cancer. Furthermore, we sought to determine if younger age is an independent prognostic factor for improved survival and analyze the safety of uterine-sparing surgery in reproductive-age women.

The finding that young women tend to have more indolent grade 1 tumours may contribute to their earlier stage at presentation and overall good prognosis. Although lower grade and earlier stage may partially explain the better survival of younger patients with ovarian cancer, younger age was an independent prognostic factor for improved survival in our multivariable analysis. Beyond conventional pathologic prognostic factors, other important molecular markers include p53 expression, Her2neu, and DNA ploidy can also help elucidate the survival differences between younger and older women (Trope *et al*, 2000; Nagai *et al*, 2001; Skirnisdottir *et al*, 2001; Chan *et al*, 2004; Nishimura *et al*, 2005; Serrano-Olvera *et al*, 2006). Studies have shown that younger patients had tumours with higher microvascular density which may be associated with an improved response to paclitaxel/platinum-based chemotherapy (Hartmann *et al*, 1994; Kosary, 1994; Chan *et al*, 2004). Our results reflect that of other studies showing that younger age is an independent prognosticator for improved survival (Yancik *et al*, 1986; Ries, 1993; Kosary, 1994; Rodriguez *et al*, 1994; Barnholtz-Sloan *et al*, 2003; O’Malley *et al*, 2003). Nevertheless, this current reports is one of the largest population-based studies that has evaluated the demographic and clinico-pathologic prognostic factors associated with the survival of younger women with ovarian cancer using detailed surgical staging and pathologic information.

Some studies have reported that younger women are able to tolerate more intensive chemotherapeutic regimens which may explain the better outcome in these patients (Thigpen *et al*, 1993; Kosary, 1994). On the other hand, case-controlled studies from single academic institutions adjusting for the experience of surgeon, extent of surgery, and adjuvant chemotherapy, younger age remained a significant independent factor for improved survival (Duska *et al*, 1999; Chan *et al*, 2003). In addition, Chan *et al* (2003) showed that younger age was an important prognostic factor for improved survival independent of age-associated determinants such as performance status. Similar findings were also reported by Thigpen *et al* (1993) who analysed a large series of patients with stage III and IV invasive epithelial ovarian cancer from six Gynecologic Oncology Group trials where strict guidelines were maintained to ensure that all patients underwent similar surgical procedures and standard adjuvant chemotherapy.

Given the large number of patients in this current study, we were able to divide our patient population into three subsets; those <30, 30–60, and >60 years. It was not surprising to find that the women <30 were more likely to have grade 1 and early-stage cancers. In fact, over 40% of ovarian cancers in the age 30–60 cohort had early-stage disease compared to less than 25% in the older cohort.

The potential role of fertility-sparing surgery in reproductive age women diagnosed with ovarian cancer has received considerable attention. In a national survey of ovarian cancer evaluating women age <25 years with ovarian malignancies, Rodriguez *et al* (1994) demonstrated that the rate of fertility-sparing surgery has increased from 46 to 70% over the 5-year study period. In this current report, we showed that uterine-sparing surgery was performed on 38.1% of reproductive age women with early-stage epithelial ovarian cancer. However, because the SEER database does not provide information regarding the extent of surgical debulking and desire to retain fertility, it is difficult to definitively validate or refute the possibilities that uterine-sparing surgery may represent suboptimally debulked patients in older women or be indicative of a desire to retain fertility in younger patients with advanced-stage disease. Nonetheless, our analyses did not find any survival difference between these women who underwent a uterine-sparing procedure compared to those that had standard surgeries in early-stage cancers (*P*=0.26). In a sub-analysis comparing women with stage I–II non-clear cell *vs* clear cell cancers, we found that there was still no survival difference based on the type of surgical procedure performed (non-clear cell epithelial cancer, *P*=0.41; clear cell, *P*=0.73). We recognise that the survival of young patients appears to have decreased over the two time periods (1988–1992; 1993–1997); however, the difference was not statistically significant (*P*=0.47) and is likely owing to the small number of patients within each group (<200). We also performed an additional analysis on the use of uterine-sparing surgeries between these two groups and identified a trend towards an increase in the use of conservative surgery over time, though, again, statistically insignificant (49.7–55.0%; *P*=0.66). Thus, it is unlikely that uterine-sparing surgeries could have contributed to the suggested findings. Although this retrospective data suggests that uterine-sparing surgeries may be considered in reproductive-age women, patients should be counselled appropriately with an understanding of the risks.

Our study was limited by the lack of information on surgeon specialty, extent of residual disease, adjuvant chemotherapy, and subsequent cytoreductive surgeries. Given that our data was derived from a nationwide cancer registry, it has shortcomings with respect to detailed clinical information such as family history, presenting symptoms, performance status, and time from symptom to presentation which may help to enhance our understanding of the underlying causes for the survival differences between the age groups. As with other large population-based series, our report was also limited by a lack of central pathology review. To determine if there are significant discrepancies between registry and referral pathologists, Piver *et al* (2000) reviewed slides from a large cancer registry and found a 95.3% complete agreement between pathologists on the disease site of origin. Moreover, there was a 61.7% complete histopathologic agreement with only 1% of cases that were considered as having major differences. Similarly, Tyler *et al* (1991) performed slide reviews on 477 women diagnosed with ovarian, breast, or endometrial cancer and compared the diagnoses of pathologists contributing to tumour registries affiliated with the SEER program to an expert panel of three gynaecologic pathologists. They found an overall agreement of 97% for overall cancers, and the agreement for major cellular subtypes of ovarian cancer was 73% for endometrioid and 100% for clear cell cancers.

The strength of this study lies in the large number of young patients with surgically staged invasive ovarian cancer, offering the ability to perform detailed, stratified analyses without sacrificing statistical strength. Furthermore, we excluded all women diagnosed with low malignant potential tumours, sarcomas, germ cell, and sex cord stromal tumours. Given that younger women are more likely to be diagnosed with borderline tumours, the inclusion of these patients may partially explain the better survival of younger women in prior studies. Additionally, this current study is one of the largest series to date of unselected patients spanning across 12 US regions, allowing for the attenuation of selection and surveillance biases often associated with clinical trials and studies from single academic institutions. Because SEER cancer registries are consistent with those for the entire country, the results from this population-based study can be generalised to the national population (Hankey *et al*, 1999). Most importantly, SEER uses several quality control measures to ensure accuracy; thus, they are able to maintain the highest level of certification of data quality and completeness as reported by the Northern American Association of Central Cancer Registries. SEER adheres to strict quality assessment measures by ensuring the accuracy of sample cases by reabstracting data from the medical records annually. Based on a recent studies by Virnig *et al* (2002), these authors found a 98% completeness in each sample case with a >90% rate in the accuracy of reporting adjuvant therapy (Virnig *et al*, 2002; Maggard *et al*, 2003). In addition, the database also appears to be accurate for major surgical procedures (Cooper *et al*, 2002).

In summary, this is one of the largest studies to date defining the status of young patients diagnosed with epithelial ovarian cancer. We found that younger patients have a better survival compared to their older cohort. Clearly, early stage and lower grade are in part responsible for the improved survival of these young women. However, after controlling for these clinico-pathologic prognostic factors, the younger group still had a better prognosis, suggests that there maybe other underlying factors such as tumour biology that can explain these findings. This analysis also suggests that reproductive-age women who undergo surgical staging should be offered conservative treatment with uterine-sparing surgeries. Moreover, given the overall encouraging outcome of younger women diagnosed with ovarian cancer, these patients need to be treated aggressively. Further research to investigate the potential biologic and molecular difference between epithelial ovarian tumours in various age groups is warranted.

## Figures and Tables

**Table 1:** Demographic data

	**Total (n=28 165)**	**Age <30 (n=400)**	**Age 30–60 (n=11 601)**	**Age >60 (n=16 164)**	***χ* ^2^**
	**n (%)**	**n (%)**	**n (%)**	**n (%)**	***P*-values**
*Age at diagnosis*					
Median (range)	64 (12–101)	26 (12–29)	50 (30–60)	72 (61–101)	
					
*Race*					
White	24 424 (86.7%)	313 (78.3%)	9698 (83.6%)	14 413 (89.2%)	<0.001^a^
Non-Hispanic	22 487 (92.1%)	258 (82.4%)	8715 (89.9%)	13 514 (93.8%)	
Hispanic	1937 (7.9%)	55 (17.6%)	983 (10.1%)	899 (6.2%)	
Black	1638 (5.8%)	31 (7.8%)	702 (6.1%)	905 (5.6%)	
Asian^b^	1496 (5.3%)	32 (8.0%)	841 (7.2%)	623 (3.9%)	
Other	607 (2.2%)	24 (6.0%)	360 (3.1%)	223 (1.4%)	
					
*Year of diagnosis*					
1988–1992	8277 (29.4%)	157 (39.3%)	3218 (27.7%)	4902 (30.3%)	<0.001
1993–1997	10 812 (38.4%)	131 (32.8%)	4416 (38.1%)	6265 (38.8%)	
1998–2001	9076 (32.2%)	112 (28.0%)	3967 (34.2%)	4997 (30.9%)	
					

a Comparing Non-Hispanic, Hispanic, Black, Asian, and Other race; when comparing White, Black, Asian, and other race, *P*<0.001.

b Includes Chinese, Japanese, Filipina, Korean, and Vietnamese.

**Table 2:** Clinico-pathologic and treatment data

	**Total (n=28 165)**	**Age <30 (n=400)**	**Age 30–60 (n=11 601)**	**Age >60 (n=16 164)**	***χ* ^2^**
	**n (%)**	**n (%)**	**n (%)**	**n (%)**	***P*-values**
*Surgical treatment*					
No surgery	5169 (18.4%)	14 (3.5%)	794 (6.8%)	4361 (27.0%)	<0.001
Uterus sparing^a^	4285 (15.2%)	208 (52.0%)	1559 (13.4%)	2518 (15.6%)	
Standard^b^	18 695 (66.4%)	178 (44.5%)	9242 (79.7%)	9275 (57.4%)	
Unknown	16 (0.1%)	0 (0.0%)	6 (0.1%)	10 (0.1%)	
					
*Stage at diagnosis*					
Stage I	6268 (22.3%)	231 (57.8%)	3639 (31.4%)	2398 (14.8%)	<0.001
Stage II	2300 (8.2%)	30 (7.5%)	1025 (8.8%)	1245 (7.7%)	
Stage III	10 113 (35.9%)	75 (18.8%)	3948 (34.0%)	6090 (37.7%)	
Stage IV	9484 (33.7%)	64 (16.0%)	2,989 (25.8%)	6431 (39.8%)	
					
*Histology*					
Epithelial	28 165 (93.1%)	400 (40.4%)	11 601 (94.0%)	16 164 (95.6%)	<0.001^c^
Non-clear cell	26 754 (88.5%)	390 (39.4%)	10 708 (86.7%)	15 656 (92.6%)	
Clear cell	1411 (4.7%)	10 (1.0%)	893 (7.2%)	508 (3.0%)	
					
*Grade of disease*					
Grade 1	2398 (8.5%)	135 (33.8%)	1391 (12.0%)	872 (5.4%)	<0.001
Grade 2	5119 (18.2%)	97 (24.3%)	2437 (21.0%)	2585 (16.0%)	
Grade 3	12 374 (43.9%)	54 (13.5%)	5059 (43.6%)	7261 (44.9%)	
Unknown grade	8274 (29.4%)	114 (28.5%)	2714 (23.4%)	5446 (33.7%)	
					

a Uterus-sparing surgeries, including minimal surgery or surgeries that did not include a hysterectomy.

b Standard surgeries, including surgeries including a hysterectomy and/or debulking.

c Compares non-clear cell epithelial with clear cell.

**Table 3:** Disease-specific 5-year survival by demography and clinico-pathology

	**Total (n=28,165)**	**Age <30**	**Age 30–60 (n=11 601)**	**Age >60 (n=16 164)**	**Log-rank**
	**%**±**s.e**.	**(*n*=400)**	**n (%)**	**n (%)**	***P*-values**
Ovarian cancer	46.1±0.4	78.8±2.4	58.8±0.6	35.3±0.5	<0.001
Stage I–II	82.9±0.5	89.7±2.1	88.8±0.5	74.5±0.8	<0.001
Stage III–IV	27.9±0.4	55.7±5.2	36.9±0.7	22.1±0.5	<0.001
					
*Race*					
White	45.7±0.4	78.6±2.6	59.2±0.6	34.9±0.5	<0.001
Non-Hispanic	45.2±0.4	79.3±2.8	59.2±0.6	34.5±0.5	<0.001
Hispanic	52.1±1.4	74.0±7.2	59.3±2.0	42.3±2.1	<0.001
Black	41.7±1.6	83.7±7.6	52.1±2.3	30.2±2.1	<0.001
Asians^a^	55.3±1.6	88.2±6.5	58.6±2.1	49.5±2.5	<0.001
					
*Year of diagnosis* b					
1988–1992	43.5±0.6	81.7±3.2	57.3±0.9	32.0±0.7	<0.001
1993–1997	46.8±0.5	74.6±4.1	58.5±0.8	37.0±0.7	<0.001
					
*Histology*					
Epithelial	46.1±0.4	78.8±2.4	58.8±0.6	35.3±0.5	<0.001
Non-clear cell	45.1±0.4	78.3±2.4	58.1±0.6	34.4±0.5	<0.001
Clear cell	65.0±1.5	100.0±0.0	67.3±1.8	60.2±2.5	=0.004
					
*Grade of disease*					
Grade 1	83.8±0.9	90.7±2.8	89.0±1.0	74.3±1.7	<0.001
Grade 2	57.1±0.8	78.5±4.7	67.2±1.1	46.2±1.2	<0.001
Grade 3	37.1±0.6	25.8±9.2	44.8±0.9	31.6±0.7	<0.001

a Includes Chinese, Japanese, Filipina, Korean, and Vietnamese.

b 1998–2001 time period not included as follow-up is not mature enough to yield 5-year survivals.

**Table 4:** Multivariate analysis

	**Hazard ratio**	**Confidence interval**	***P*-value**
Stage of disease^a^	1.93	(1.89–1.97)	*P*<0.001
Histologic cell type^b^	1.27	(1.15–1.40)	*P*<0.001
Age at diagnosis^c^	1.02	(1.02–1.02)	*P*<0.001
Grade of disease^d^	1.02	(1.01–1.03)	*P*<0.001
Year of diagnosis^c^	0.99	(0.98–0.99)	*P*<0.001
Surgical treatment^e^	0.69	(0.68–0.71)	*P*<0.001
Race/Ethnicity^f^	0.99	(0.96–1.02)	*P*=0.71

a Stage I *vs* Stage II *vs* Stage III *vs* Stage IV.

b Non-clear cell epithelial cancer *vs* clear cell cancer.

c As a continuous variable.

d Grade 1 *vs* Grade 2 *vs* Grade 3 *vs* unknown Grade.

e No surgery *vs* any surgery.

f White *vs* Black *vs* Asians *vs* Others.
